# Identification of a Distinct miRNA Regulatory Network in the Tumor Microenvironment of Transformed Mycosis Fungoides

**DOI:** 10.3390/cancers13225854

**Published:** 2021-11-22

**Authors:** Cosimo Di Raimondo, Zhen Han, Chingyu Su, Xiwei Wu, Hanjun Qin, James F. Sanchez, Yate-Ching Yuan, Xochiquetzal Martinez, Farah Abdulla, Jasmine Zain, Chun-Wei Chen, Steven T. Rosen, Christiane Querfeld

**Affiliations:** 1 Division of Dermatology, City of Hope, Duarte, CA 91010, USA; cosimodiraimondo@gmail.com (C.D.R.); zhan@coh.org (Z.H.); csu@coh.org (C.S.); xumartinez@gmail.com (X.M.); Fabdulla@carisls.com (F.A.); 2 Department of Dermatology, University of Roma Tor Vergata, Rome 00133, Italy; 3 Beckman Research Institute, City of Hope, Duarte, CA 91010, USA; jamsanchez@coh.org (J.F.S.); cweichen@coh.org (C.-W.C.); srosen@coh.org (S.T.R.); 4 Department of Molecular and Cellular Biology, City of Hope, Duarte, CA 91010, USA; XWu@coh.org (X.W.); HQin@coh.org (H.Q.); 5 Integrative Genomics Core, City of Hope, Duarte, CA 91010, USA; 6 Department of Hematology and Hematopoietic Cell Transplantation, City of Hope, Duarte, CA 91010, USA; jazain@coh.org; 7 Department of Computational Quantitative Medicine, City of Hope, Duarte, CA 91010, USA; YYuan@coh.org; 8 Translational Bioinformatics, Center for Informatics, City of Hope, Duarte, CA 91010, USA; 9 Department of Systems Biology, City of Hope, Duarte, CA 91010, USA; 10 Department of Pathology, City of Hope, Duarte, CA 91010, USA

**Keywords:** cutaneous T-cell lymphoma (CTCL), mycosis fungoides (MF), large cell transformation of mycosis fungoides (LCT-MF), miR regulatory network, tumor immune microenvironment

## Abstract

**Simple Summary:**

Transformed mycosis fungoides (LCT-MF) is a histopathological marker of poor prognosis and associated with worse survival. We compared miRNA and mRNA expression profiles of LCT-MF with classic MF and found a distinct miRNA regulatory network modulated immunosuppressive tumor microenvironment in LCT-MF. Our findings provide novel insights and therapeutic targets for LCT-MF.

**Abstract:**

Large cell transformation of mycosis fungoides (LCT-MF) occurs in 20–50% of advanced MF and is generally associated with poor response and dismal prognosis. Although different mechanisms have been proposed to explain the pathogenesis, little is known about the role of microRNAs (miRs) in transcriptional regulation of LCT-MF. Here, we investigated the miR and mRNA expression profile in lesional skin samples of patients with LCT-MF and non-LCT MF using RNA-seq analysis. We found miR-146a and miR-21 to be significantly upregulated, and miR-708 the most significantly downregulated miR in LCT-MF. Integration of miR and mRNA expression profiles revealed the miR-regulated networks in LCT-MF. Ingenuity pathway analysis (IPA) demonstrated the involvement of genes for ICOS-ICOSL, PD1-PDL1, NF-κB, E2F transcription, and molecular mechanisms of cancer signaling pathways. Quantitative real time (qRT)-PCR results of target genes were consistent with the RNA-seq data. We further identified the immunosuppressive tumor microenvironment (TME) in LCT-MF. Moreover, our data indicated that miR-146a, -21 and -708 are associated with the immunosuppressive TME in LCT-MF. Collectively, our results suggest that the key LCT-MF associated miRs and their regulated networks may provide insights into its pathogenesis and identify promising targets for novel therapeutic strategies.

## 1. Introduction

Cutaneous T cell lymphoma (CTCL) is a disfiguring, incurable malignancy with a poor prognosis for those with advanced stage disease. Mycosis fungoides (MF) is the most common primary CTCL and is characterized by expansion of malignant T-cells in a chronic inflammatory environment in the skin [1]. MF evolves through patches and plaques to tumors and/or erythroderma with or without systemic involvement. Patients with early-stage disease have a 10-year survival ranging between 64–98%, whereas patients with advanced stage disease have a less favorable prognosis, with a 10-year survival of 20–42% [2,3]. Large cell transformation (LCT) of CTCL, histopathologically defined by sheets or microscopic nodules of atypical T cells with a large cell phenotype four times larger than normal lymphocytes that make up at least 25% of the dermal infiltrate, is associated with rapidly progressing disease and dismal prognosis and confers a median survival of 1–4 years after transformation, which is not overcome by any regimen to date [4,5,6].

Several efforts have been made to identify biomarkers of transformation that could help prompt an early and targeted treatment to lead to a more favorable clinical outcome. The most promising targets of these investigations are the genetic aberrations and epigenetic changes that have been associated with disease progression and survival in patients with MF [7]. Notably, many genetic and chromosomal abnormalities have been identified in the pathogenesis and progression of MF, such as amplification of JUNB, which is involved in T-cell proliferation, differentiation, and apoptosis, [8,9] and activation of the STAT family transcription factors [10]. Furthermore, decreased expression of regulating proteins p14, p15, and p16, which can interact with cyclin-dependent kinases and induce cell cycle arrest, has been described in MF [11]. Nevertheless, these genetic and chromosomal abnormalities are not sufficient to predict the outcome of LCT-MF and additional analyses are critically needed to better dissect the molecular basis of transformation.

MicroRNAs (miRs) are a class of short noncoding RNAs that regulate gene expression. They are involved in physiological processes and disease development and have an important role in DNA damage response [12]. Dysregulated miRNAs have been linked to cancer, acting either as oncogenes or tumor suppressors, and appear to be involved in the pathogenesis and progression of CTCL [13,14]. In MF, several studies have identified upregulated miR-155 in cutaneous lesions of MF, which has led to the clinical development of a miR-155 inhibitor in CTCL and other hematological malignancies [15]. The upregulation of miR-155 with concomitant downregulation of miR-203 and miR-205 in MF was found to be useful to discriminate MF from benign dermatoses [16]. Notably, Lindahl et al. [17] developed a 3-miR classifier, based on miR-106b-5p, miR-148a-3p, and miR-338-3p, that appears to predict disease progression of early MF, and Manso et al. demonstrated that miRs regulate MF progression [18]. MiR expression profiling has also been used for prognostication in tumor stage and subtypes of MF; high expression of miR-155 and low expression of miR-200b correlated with worse prognosis [13]. However, the mechanisms by which the miRs contribute to the signaling pathways and microenvironmental immune cell profiles in LCT-MF remain unknown. The goal of this study was to identify differentially expressed miRs, the regulatory network targeting pathways of LCT-MF, and their effects on the TME, providing insights into pathogenesis and potentially identifying promising targets for novel therapeutic strategies in LCT-MF.

## 2. Materials and Methods

### 2.1. Patients and Tissue Samples

A prospective consecutive case-series of 28 patients (20 males, 8 females; median age: 63.6 years, range: 30.8–85.4 years) diagnosed with MF received little to no treatment prior to their initial visit in our multidisciplinary cutaneous lymphoma clinic. Subjects had signed informed consent for specimen collection for research purposes, in accordance with the Declaration of Helsinki and approval by the City of Hope Institutional Review Board. Patients were diagnosed according to the revised 2018 WHO-EORTC classification [2] and classified according to the revised staging system for MF [19]. Table 1 summarizes patient characteristics. Skin disease was classified according to T stage. T1 and T2 are defined by plaques involving <10% and ≥10% of the body surface area, respectively, whereas T3 is defined by ≥1 tumor and T4 by erythroderma. Relevant demographic and clinical information was collected from the electronic medical records including age, gender, race/ethnicity, diagnosis, stage, histopathologic findings such as transformation status, and assessment of the skin tumor burden by the severity-weighted assessment tool (mSWAT). LCT-MF is defined as showing large cells (4 times the size of a small lymphocyte) in 25% or more of the dermal infiltrate or forming microscopic nodules [6].

### 2.2. RNA Sequencing and Library Preparation

Total RNA from 34 paraffin-embedded (FFPE) lesional CTCL skin biopsy sections (size uniform, plaques and tumors ≥ 1 cm^2^), which included 14 samples of large cell transformation (7 plaques, 7 tumors) and 20 non-transformed lesions (12 plaques, 8 tumors) was extracted with a miRNAeasy FFPE kit (Qiagen, Valencia, CA, USA). Library preparation and RNA sequencing were performed as described in our previous publication [20]. Briefly, 300 ng of the total RNA isolation was used. Small RNA library preparation was performed following the Illumina protocol with minor modifications. The library was created from each RNA sample by 3′ adapter ligation, 5′ RT primer annealing, 5′ adapter ligation, reverse transcription, and PCR amplification. Sequencing of 50 cycles was performed on a HiSeq 2500 (Illumina, San Diego, CA, USA). Demultiplexing of the raw sequencing data and generation of the fastq files were performed using the Real-Time Analysis (RTA) software. For Ribo-Zero RNA Sequencing, total RNA was rRNA-depleted using the Ribo-Zero low input kit for Human/Mouse/Rat (Kapa Biosystems, Wilmington, NC, USA). RNA-seq libraries were prepared from Ribo-Zero mRNA-enriched material using KAPA Stranded RNA-Seq Library Preparation Kit (Illumina Platforms) (Kapa Biosystems, Wilmington, NC, USA), using 10 cycles of PCR amplification. Libraries were purified using AxyPrep Mag PCR Clean-up kit (Thermo Fisher Scientific, Irwindale, CA, USA). Sequencing was performed on an Illumina^®^ Hiseq 2500 (Illumina, San Diego, CA, USA) instrument using the TruSeq SR Cluster Kit V4-cBot-HS (Illumina^®^) to generate 51 bp single-end reads sequencing with v4 chemistry. Quality control of RNA-Seq reads was performed using FastQC.

### 2.3. Sequencing Data Analysis

miR and mRNA-seq data were analyzed as previously described [20] with the following modification: The mapping table was created using the human miR mature sequences from miRBase release18 and aligned back to human hg19 genome afterwards. Differential expression analysis was conducted using Bioconductor package “edgeR”, with *p* value < 0.01 and fold change > 1.5 as the cutoff values for miRNA data. For RNA-seq data, “limma” was used on log2 transformed RPKM values, with *p* value < 0.05 and fold change > 1.5 as the cutoff values. Hallmark pathway enrichment analyses were carried out using gene set enrichment analysis (GSEA) with genes sorted by—log10 (*p* value) with sign determined by the fold change between MF-LCT and non-LCT samples. An FDR ≤ 0.05 and the involvement of at least 2 genes were used as the cutoff criteria. These data sets are deposited in the Gene Expression Omnibus (GEO) repository under the accession numbers GSE181130 and GSE113113.

### 2.4. Quantitative Real Time (qRT)-PCR Validation

Validation of miRs and mRNA expression was performed using qRT-PCR. miR analysis was performed using TaqMan™ MicroRNA Reverse Transcription Kit (cat#: 4366596, Thermo Fisher Scientific) and TaqMan™ Fast Advanced Master Mix (cat#: 4444963, Thermo Fisher Scientific). miR expression was normalized by a reference smRNA (RNU48) gene. mRNA analysis was performed using a High-Capacity cDNA Reverse Transcription Kit (cat#: 4368814, Thermo Fisher Scientific) and TaqMan™ Fast Advanced Master Mix (cat#: 4444963, Thermo Fisher Scientific). mRNA expression was normalized to OAZ1 as a reference gene. The primers used in these analyses were listed as follows: hsa-miR-146a-3p (Assay ID: 002163, Thermo Fisher Scientific), hsa-miR-21-3p (Assay ID: 002438, Thermo Fisher Scientific), hsa-miR-708-5p (Assay ID: 002341, Thermo Fisher Scientific), RNU48 (Assay ID: 001006, Thermo Fisher Scientific), STAT3 (Hs00374280_m1, Thermo Fisher Scientific), NR4A2 (Hs00428691_m1, Thermo Fisher Scientific), NF-κB p65 (Hs00153294_m1, Thermo Fisher Scientific), TGFβ1 (Hs00998133_m1, Thermo Fisher Scientific), E2F1 (Hs00153451_m1, Thermo Fisher Scientific), ICOSLG (Hs00323621_m1, Thermo Fisher Scientific), CDK1 (Hs00938777_m1, Thermo Fisher Scientific), MYC (Hs00153408_m1, Thermo Fisher Scientific), PDCD1 (Hs01550088_m1, Thermo Fisher Scientific), PD-L1 (Hs00204257_m1, Thermo Fisher Scientific), ICOS (Hs00359999_m1, Thermo Fisher Scientific), OAZ1 (Hs00427923_m1, Thermo Fisher Scientific). PCR reactions for each sample were carried out in triplicates and 2^ (-delta delta CT) value was performed for statistical analysis.

### 2.5. Functional Gene Set Enrichment Analysis

An interaction network of mRNAs in LCT-MF that are upregulated and associated with miR-146a and -21 was generated using Ingenuity Pathway Analysis (IPA, Ingenuity^®^ Systems, www.ingenuity.com, accessed on 10 October 2020) with fold change > 1.5, FDR < 0.05.

### 2.6. Multiplex Immunofluorescence Staining

FFPE tissue sections slides at 5 µm thickness were subjected to multiplex immunofluorescence staining with antibodies against the following: CD4 (1:300, SP35; Cell Marque, Rocklin, CA, USA), CD8 (1:50, SP16; Biocare Medical, Pacheco, CA, USA), PD-1 (1:3000, UM800091 Origene, Rockville, MD, USA) and PD-L1 (1:400, SP142; Abcam, Burlingame, CA, USA) as previously described [20]. Multispectral images were acquired using a Vectra 3 microscope (Vectra AI, San Jose, CA, USA), and different channels were separated with QUPATH v0.2.0-m9.

### 2.7. Statistical Analysis

Kaplan–Meier survival curves with Mantel–Cox test was used for survival analysis. Student’s *t* test was used to analyze the data when not otherwise specified. All *p* values were two-sided, and the statistical significance level was set at *p* < 0.05. Correlation between differentially expressed miRs and immune cell gene score was analyzed by Pearson’s correlation coefficient using Bioconductor package “edgeR”. Statistical analyses were carried out using Prism 8.2.0 (GraphPad, La Jolla, CA, USA) when not otherwise specified.

## 3. Results

### 3.1. Outcomes for Patients with LCT-MF Are Discouraging

**Figure 1:**
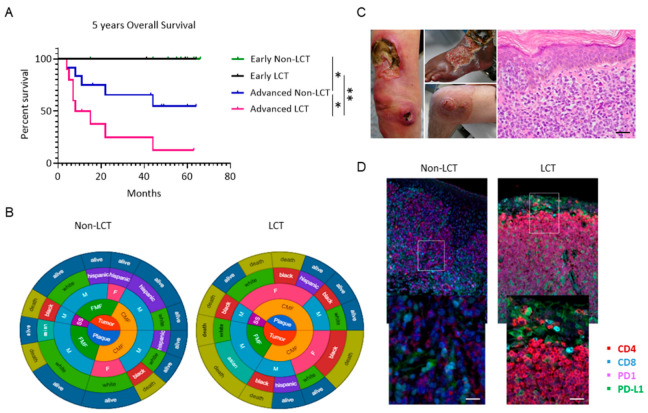
Kaplan–Meier analysis shows significantly decreased survival of patients with LCT-MF compared to non-LCT-MF. (**A**) The overall survival of early non-LCT (green) or early LCT-MF (purple) patients was compared to advanced non-LCT (blue) or advanced LCT-MF patients (magenta) and determined by Mantel–Cox test with *p* value < 0.05 considered significant (*n* = 28). * *p* < 0.05, ** *p* < 0.01. (**B**) Circular charts show the distribution of demographic characteristics including stage, MF subtype, gender, race, and survival status of LCT and non-LCT patients. (**C**) Clinical presentation of patients with LCT-MF and Hematoxylin and Eosin (H&E) staining of the LCT-MF lesional skin. Scale bar = 50 μm. (**D**) Representative multiplex immunofluorescence images of CTCL skin biopsy with LCT-MF and non-LCT-MF. CD4 is stained red; CD8 is blue; PD1 is magenta; PD-L1 is green. Scale bar = 20 μm.

Clinical stage is an important determinant of risk for disease progression (RDP) and overall survival (OS) [3]. The clinical characteristics and distribution of patients are summarized in Table 1. Twenty-eight patients diagnosed with MF (20 males and 8 females) with a median age of 63.6 years (range 30.8–85.4) were consecutively enrolled in the study. Eleven patients had LCT-MF and 17 had non-LCT. Among non-LCT patients, approximately 50% of patients had early-stage disease (IA–IB), and 50% had advanced stage disease (IIB, IVA), whereas 73% of LCT-MF patients were diagnosed with advanced stages (IIB, IVA). Folliculotropic MF was higher in the non-LCT group (35.3%) than in the LCT-MF group (18.2%). LCT-MF was predominantly seen in African Americans and in advanced stages, presenting with cutaneous tumor lesions and/or extracutaneous disease, in contrast to non-LCT. At the time of data analysis, 9 patients had died, and 19 patients were censored by their last appointment. Assessment of the overall survival (OS) in the entire patient cohort revealed that patients with advanced LCT-MF had a significantly decreased OS compared to patients with non-LCT (*p* = 0.039); our clinical analysis showed a 2-year OS of 80% for advanced classic MF versus 14.3% in patients with advanced LCT-MF, *p* = 0.0098 (Figure 1A). Two circular charts illustrate the demographic, clinical and survival characteristics of LCT-MF and non-LCT MF patients and highlight a preponderance of female black patients with advanced stage in the LCT cohort and of white males in the non-LCT cohort, as well as a higher presentation of folliculotropic type of MF (Figure 1B). Most patients have died of disease progression in the LCT-cohort, highlighting the dismal prognosis. Clinical presentations of patients with LCT-MF characterized by ulcerated tumor lesions are seen in Figure 1C. On histopathology, an atypical epidermotropic lymphoid infiltrate with large cell morphology is noted. The large pleomorphic cells uniformly show high expression for PD1 and PD-L1 (Figure 1D).

### 3.2. The miR Expression Profile in LCT-MF Is Distinct from That of Non-LCT

**Figure 2:**
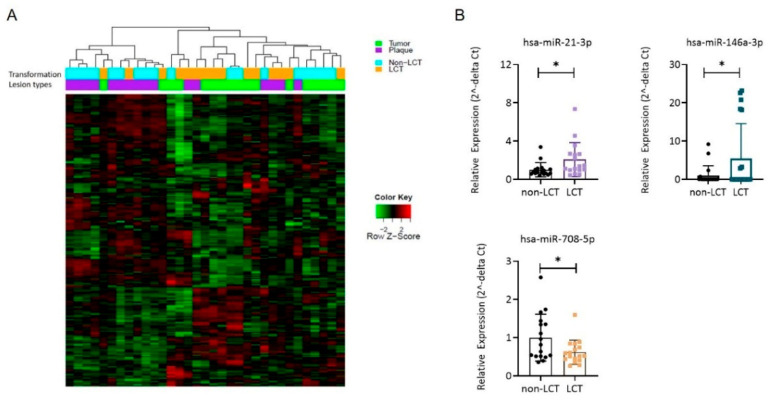
miR signatures in LCT-MF. (**A**) Heatmap of unsupervised two-way hierarchical clustering based on global miR analysis. (**B**) qRT-PCR-based analysis of miR expression in LCT-MF and non-LCT lesional skin samples. The 2ˆ (-delta delta CT) method was used as a relative quantification strategy for data analysis. Results are shown as means ± SD, and differences were tested for significance using Student’s *t* test (* *p* < 0.05).

We determined the miR profiles from skin biopsies of 19 LCT-MF and 15 non-LCT patients using small RNA sequencing. The unsupervised heatmap of miR analysis reveals different miR clusters for LCT and non-LCT-MF (Figure 2A). Furthermore, comparison of the miR expression levels in LCT-MF plaques and tumors with that in non-LCT-MF identified 17 differentially expressed miRs with an adjusted *p* value < 0.05 (Table 2). The highest-ranked miRNAs included miR-21-3p (abbreviated as miR-21 below), miR-146a-3p (abbreviated as miR-146a below), miR-136-5p, miR-889 and miR-539-3p. The majority (seven miRs) were downregulated, with a log fold change (log FC) < −2. The top downregulated miRs included miR-708-5p, miR-5701, and miR-3653, which are widely viewed as tumor suppressor miRs and noted in many cancers during tumor progression. The upregulation of miR-21 and miR-146a and downregulation of miR-708 in LCT-MF were verified by qRT-PCR analysis. As shown in Figure 2B, both miR-146a (*p* < 0.05) and -21 (*p* < 0.05) expression levels were found to be elevated and miR-708 (*p* < 0.05) was decreased in LCT-MF, validating the findings of RNA-seq analysis. Thus, these results suggested that miR-146a and -21 might be critical for LCT-MF progression.

### 3.3. MRNA Expression Profiling Distinguishes LCT-MF from Non-LCT MF

**Figure 3:**
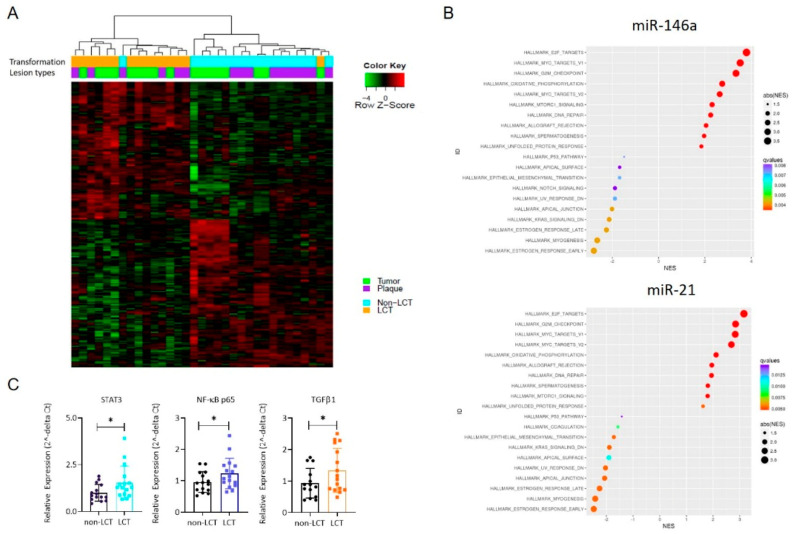
Transcriptional profiles of LCT-MF and non-LCT MF. (**A**) Unsupervised clustering of mRNA seq data of plaques and tumor lesions of LCT-MF segregated from non-LCT MF. (**B**) Hallmark pathway analysis of genes that correlate with miRNA-21 and miR-146a. (**C**) qRT-PCR-based analysis of mRNA expression in MF-LCT and non-LCT lesional skin samples. The data are presented as means ± SD. Differences between means were analyzed using Student’s *t* test (* *p* < 0.05).

We compared the global mRNA expression profile of LCT vs. non-LCT cases chosen by histopathologic criteria. Hierarchical clustering revealed two distinct clusters segregated by transformation status, shown in the unsupervised mRNA heatmap (Figure 3A). The left cluster reveals LCT-MF cases (plaques and tumors); the right cluster identifies non-LCT MF. To identify the potential functions of the identified differentially expressed genes (DEGs), we performed Hallmark pathway analysis. The top enriched Hallmark pathway terms for both miRs included E2F targets, MYC targets, G2M checkpoint, oxidative phosphorylation, DNA repair and MTORC1. The minimally enriched and negatively enriched Hallmark pathway terms among DEGs are shown in Figure 3B. qRT-PCR was performed to verify the expression patterns of mRNAs determined by RNA seq. As shown in Figure 3C, the findings showed that the expression patterns of *STAT3* (*p* < 0.05), *NF-κB p65* (*p* < 0.05) and *TGFβ1* (*p* < 0.05) were consistent with the RNA-seq data. These findings indicated that in most cases the qRT-PCR results were consistent with those of RNA-seq, which implied that the mRNA expression profile in LCT-MF is reliable.

### 3.4. Identification of a Functional Interaction Network of Upregulated Genes in LCT-MF That Positively Correlated with miR-146a and miR-21

**Figure 4:**
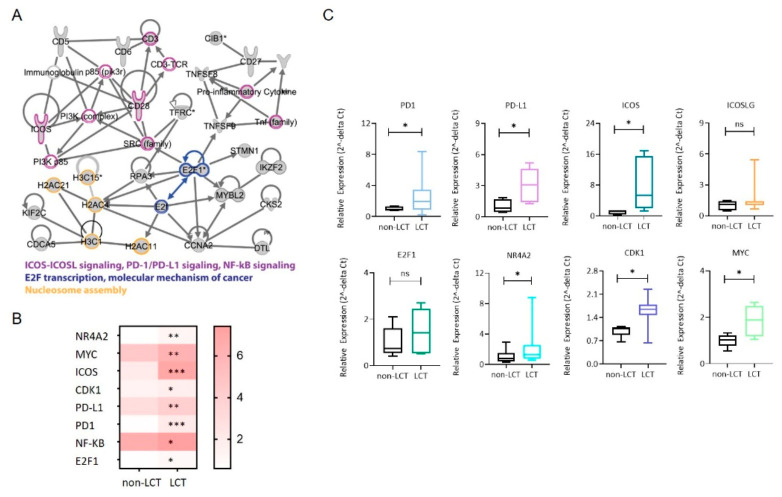
Identification of signaling network of upregulated mRNAs that positively correlate with miR-21 and miR-146a. (**A**) Functional interaction network of upregulated genes in LCT-MF. The 130 genes that are upregulated in LCT-MF and positively associated with miR-146a and miR-21 were subjected to network analysis using Ingenuity Pathway Analysis (IPA). The nodes are colored based on their associated canonical pathways. (**B**) Heatmap visualization of differential expression key genes in MF-LCT vs. non-LCT. * *p* < 0.05, ** *p* < 0.01, *** *p* < 0.001. (**C**) qRT-PCR validation of key genes of the targeted signaling in LCT-MF and non-LCT lesional skin samples. Data are shown as means ± SD. Variables were analyzed using Student’s *t* test (* *p* < 0.05); ns, no significant difference.

To further demonstrate the functional impact of miR-146a and miR-21, we also used functional analysis tools of Ingenuity Pathway Analysis (IPA) to analysis DEGs. There were 49 upregulated genes (including *STAT3* and *NF-κB*) that positively correlated with miR-21 and 116 upregulated genes that positively correlated with miR-146a. IPA demonstrated the involvement of relevant genes in pathways of ICOS-ICOSL signaling, PD1-PDL1 signaling, NF-κB signaling, E2F transcription and molecular mechanisms of cancer (Figure 4A). RNA sequencing of the LCT-MF and non-LCT MF lesional skin revealed that among the activated pathways regulating large cell transformation, the expression of the genes *NR4A2* (*p* < 0.01), *MYC* (*p* < 0.01), *ICOS* (*p* < 0.001), *CDK1* (*p* < 0.05), *PD-L1* (*p* < 0.01), *PD1* (*p* < 0.001), *NF-κB* (*p* < 0.05), and *E2F1* (*p* < 0.05) were significantly upregulated in MF-LCT (Figure 4B). Furthermore, we validated the key genes in the targeted pathways using qRT-PCR in MF-LCT versus non-LCT. As shown in Figure 4C, the results revealed that the gene expression trends of *PD1* (*p* < 0.05)*, PD-L1* (*p* < 0.05)*, ICOS* (*p* < 0.05)*, ICOSLG* (ns), *E2F1* (ns), *NR4A2* (*p* < 0.05), *CDK1* (*p* < 0.05), and *MYC* (*p* < 0.05) were consistent with the RNA-seq data. These results suggest that these pathways are involved in mediating the effects of miRNAs in LCT-MF.

### 3.5. Distinct Microenvironmental Immune Cell Populations in LCT-MF

**Figure 5:**
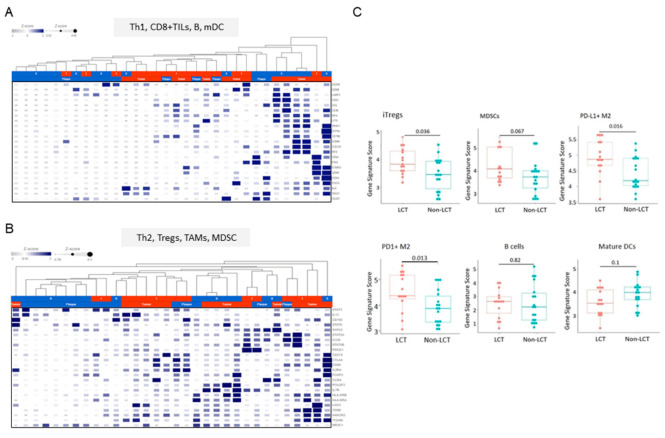
Immune cell gene signatures in the microenvironment of LCT-MF vs. non-LCT. Heatmap visualization of RNA-Seq data: Th1, CD8+ TILs, and B cells (**A**) Th2, Tregs, TAMs, MDSCs and DCs signature (**B**) in the microenvironment of MF-LCT vs. non-LCT. (**C**) RNA-Seq analysis with genes scored by −log2 (RPKM) to evaluate the genetic expression of iTregs, MDSCs, PD-L1+ M2, PD1+ M2, B cells, and mature DCs in MF-LCT vs. non-LCT. “limma” was used with *p*-value < 0.05 and fold change > 1.5 as the cutoff values.

To understand the diversity of immune cell types that drive LCT-MF pathogenesis, we profiled microenvironmental immune cell gene signatures with CIBERSORT. The analysis of immune cell abundance estimated using CIBERSORT identified signatures for naïve CD4+ T cells (*p* < 0.01), resting dendritic cells (*p* < 0.001), gamma delta T cells (*p* < 0.05), M0 macrophages (*p* < 0.05), M2 macrophages (*p* < 0.05) as more abundant, whereas CD4+ T cells (*p* < 0.001) and mast cells were less abundant in LCT-MF than in non-LCT (Figure S1A). When comparing the PD1 and PD-L1 transcripts in LCT-MF with those in non-LCT, we found a higher frequency of PD1 transcripts in CD8+ T cells and CD4+ T cells in LCT-MF than in non-LCT-MF and PD1 transcripts in neutrophils in non-LCT over that in LCT; a higher frequency of PD-L1 transcripts were detected in B cells, CD8+ T cells, and eosinophils in non-LCT than in LCT, whereas neutrophil expression was relatively higher in LCT (Figure S1B). Furthermore, sequencing analysis was performed using gene sets representing B cells, CD8+ tumor-infiltrating lymphocytes (TILs), tumor-associated macrophages (TAMs), dendritic cells (DC), myeloid-derived suppressor cells (MDSC), regulatory T cells (Tregs), and Th1/Th2 cell memory phenotypes to analyze the immune cell gene profile in the CTCL microenvironment of LCT-MF versus non-LCT. Heatmaps were generated using the defined genes representing immune cell types (Figure 5A,B). We used the Kruskal–Wallis test to assess the average expression scores of relevant immune cell related genes including TAMs (M2), MDSCs, iTregs, B cells, and mature DCs stratified by transformation status (Figure 5C). Our data indicate that microenvironmental immune cell gene profiling in LCT-MF demonstrated a significant elevated gene signature for inducible (i) Tregs (*p* = 0.036), PD-L1+ M2 macrophages (*p* = 0.016), and PD1+ M2-macrophages (*p* = 0.013) and a trend for elevated genes for MDSCs (*p* = 0.067) and for a decreased gene signature for mature DCs (*p* = 0.1), which may indicate an immune cell profile associated with immunosuppression and progression of LCT. There was no significant difference seen in B cell -related genes (*p* = 0.82) between LCT-MF vs. MF non-LCT. The findings indicate an immunosuppressive TME in LCT-MF.

### 3.6. MiR Expression Correlates with the Immune Cell Gene Score in LCT-MF

**Figure 6:**
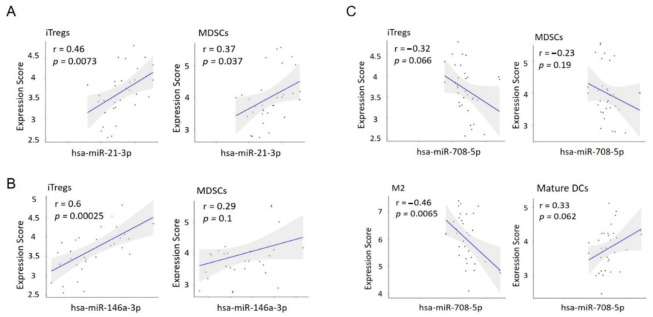
The correlation analysis of miR expression and the immune cell gene score in MF-LCT. (**A**) Correlation analysis of miR-21-3p with iTregs and MDSCs in MF-LCT. (**B**) Correlation analysis of miR-146a-3p with iTregs and MDSCs in MF-LCT. (**C**) Correlation analysis of miR-708-5p with iTregs, MDSCs, TAM (M2) and immature DCs in MF-LCT. Pearson’s correlation coefficient.

Lastly, we wanted to investigate the relationship between miR expression and the immune cell gene signature in LCT-MF. To this end, a Pearson’s correlation coefficient analysis was carried out between miR expression and the average gene score of immune cells. miR-21, which is highly expressed in LCT-MF, showed a strong positive correlation with iTreg (r = 0.46, *p* = 0.0073) and MDSC (r = 0.37, *p* = 0.037) gene expression (Figure 6A); the upregulated miR-146a also positively correlated with iTreg gene score (r = 0.6, *p* = 0.00025) in LCT-MF (Figure 6B). Although not significant, there was a trend toward a positive correlation with MDSCs. Moreover, the downregulated miR-708 negatively correlated with the TAM (M2) gene score (r = 0.46, *p* = 0.0065; Figure 6C), suggesting miR-708-mediated effects could inhibit an immunosuppressive environment, through inhibition of TAM (M2) gene expression. A trend was also seen for negative correlation with iTregs and MDSCs and a positive correlation with mature DCs. In summary, upregulated miR-21 and miR-146a and downregulated miR-708 drive an immunosuppressive tumor microenvironment that may contribute to the transformed phenotype in MF and impact tumor growth.

## 4. Discussion

The role of epigenetic dysregulation in the pathogenesis of LCT-MF is an area of active research and clinical interest. Notably, a phase 1 study of MRG-106 (cobomarsen), an inhibitor of miR-155, has shown efficacy in MF patients. The role and regulation of miR profiles in LCT-MF pathogenesis has not previously been explored. Therefore, the identification of the miRs targeting regulatory signaling pathways and their effects on the immune microenvironment in LCT-MF is critical to develop effective treatment strategies.

Previous studies have identified LCT as an independent prognostic factor in patients with MF, portending a poor prognosis and outcome [21]. Our findings confirm previously published data [21,22] highlighting a dismal 2-year OS of 14.3% in patients with LCT-MF versus 80% for non-LCT MF. Transformation occurs predominantly in advanced/progressing stages of MF presenting with cutaneous and/or extracutaneous tumors. However, in our cohort, a subset of early stage LCT-MF, clinically presenting with cutaneous plaque, was included. We found 12 miRNAs (five upregulated and seven downregulated) differentially expressed in LCT-MF tumors and plaques compared to non-LCT MF. The signatures in plaques were similar to tumors and may indicate early prognostic factors for transformation and progression. In non-LCT-MF, we had recently established that the positive correlation of miR-155, -130, and-21 with an immune checkpoint (IC) expression profile provides justification for miRs as a putative therapeutic target to reverse T cell exhaustion in CTCL [23]. To our surprise, the upregulated miRs in LCT-MF cases did not include miR-155. Although data show that miR-155 is linked to the oncogenesis of MF, with higher expression noted in tumor lesions [24], our findings suggest that miR-155 regulation is not critical for transformation. Among the upregulated miRs in LCT-MF, we found that the miR with the highest fold difference was miR-146a. MiR-146a has emerged as key regulator of immune responses and tumorigenesis in various cancers [25,26]. MiR-21 was previously identified as the top related miR associated with disease progression and as highly upregulated in advanced CTCL stages [27]. Lindahl LM et al. found miR-21 expression was driven by JAK3/STAT5 in both malignant and non-malignant T cells in MF [28].

To date, miR-regulated signaling pathways remain largely unknown in LCT-MF. In this study, we identified the upregulated miR-related signaling network in LCT-MF including ICOS-ICOSL signaling, PD1-PDL1 signaling, NF-κB signaling, E2F transcription, and molecular mechanisms of cancer pathways. Although these pathways have been consistent with previous studies in MF, other specific pathways of LCT-MF in this analysis, such as E2F transcription, have not been previously reported and are worthwhile to explore, as the interaction of malignant cells with E2F, for one, promotes a microenvironment suitable for tumor cell growth, survival, and aggressiveness [29,30]. Among the downregulated miRs, the miR with the most statistically significant log FC was the hsa-miR-708. There are opposing results among studies as to the oncogenic or tumor suppressor function of miR-708, which may be due to the identity of targets driving various cancers [31]. Little is known in T cell malignancies. Low miR-708 expression was found to correlate with high-risk T cell progenitor-derived T-ALL and poor prognosis [32]. Notably, miR-708 may function as a tumor suppressor by directly targeting the innate checkpoint CD47 in T-ALL [33]. Restoration of miR-708 expression in a T-ALL cell line promoted phagocytosis by macrophages via downregulation of CD47 to eliminate T-ALL cells. Considering the emerging role of miRs involved in immune responses, these miR candidates seem to be of great interest for further exploration in LCT-MF.

The importance of the cellular microenvironment in various solid and hematologic malignancies including CTCL has been increasingly recognized [3,34,35]. We sought to identify the effects of miRs on the immune cell gene profile in MF-LCT. A significant gene score was noted for iTregs and PD-L1+ M2 in MF-LCT when compared to non-LCT, suggesting that iTregs and PD-L1+ M2 maybe the most abundant cell types in the tumor microenvironment in localized LCT-MF. Although not statistically significant, possibly due to the overall small sample size, there was a trend toward statistically significant increased TAM (M2) MDSC and B cell gene scores in LCT-MF and a decreased mature DC gene score. Tumor-derived miRs may mediate intercellular communication affecting the maturation status of DCs. Several studies demonstrated a decrease in DC subsets and impaired cell-mediated immunity in MF [36,37]. Manso et al. found that miR-146a was highly expressed in advanced stages of MF and directly targeted the *FOXP3* gene, involved in Treg differentiation and MF progression [18]. We and others have shown the pro-tumorigenic role of TAM2-macrophages in MF [38]. In line with these observations, our findings support an immunosuppressive TME in LCT-MF. Recent studies have shown that miRs play a role in remodeling the TME [39,40]; however, it remains unknown which miRs regulate the immunosuppressive microenvironment in LCT-MF. We identified specific miR signatures associated with immune cell mRNA signatures. Notably, upregulated miR-21 and miR-146a positively correlated with iTreg gene score, and downregulated miR-708 negatively corelated with TAM (M2) gene score in MF-LCT. These findings show that not only the upregulated miRs, but also the downregulated miRs, promote the formation of an immunosuppressive microenvironment in LCT-MF.

## 5. Conclusions

In conclusion, our study identified miR-regulated signaling networks and their effects on immune cell gene profiles in LCT-MF, which may prove relevant to pathogenesis and hold promise for the identification of potential therapeutic targets in this disease.

## Figures and Tables

**Table 1:** Clinical features of 28 patients with mycosis fungoides with and without large cell transformation (there is little or no prior past therapies).

Characteristic	Non-LCT	LCT
Patients/Skin biopsy specimens	17/20	11/14
Age (years), median (range)	58.4 (30.8–85.4)	68 (41.3–82.8)
Gender		
Male	14 (82.4%)	6 (54.5%)
Female	3 (17.6%)	5 (45.5%)
Race/Ethnicity		
Caucasian	8 (47.1%)	3 (27.3%)
African American	2 (11.8%)	6 (54.5%)
Hispanic	5 (29.4%)	1 (9.1%)
Asian	2 (11.8%)	1 (9.1%)
Clinical stage		
IA	3 (17.6%)	0
IB	5 (29.4%)	3 (27.3%)
IIB	8 (47.1%)	5 (45.5%)
IVA	1 (5.9%)	3 (27.3%)
MF histologic subtype		
Classic	11 (64.7%)	9 (81.8%)
Folliculotropic	6 (35.3%)	2 (18.2%)

LCT, large cell transformation; MF, mycosis fungoides.

**Table 2:** Up- and downregulated miRNAs MF-LCT vs. non-LCT.

Genes	Log FC	Log CPM	F	*p* Value	lFDR	Non-LCT	LCT	Status
hsa-miR-146a-3p	1.71	4.97	11.81	0.001717	0.174221	16	49	Up
hsa-miR-21-3p	1.05	7.56	10.12	0.003353	0.202974	105	288	Up
hsa-miR-136-5p	0.89	5.21	9.07	0.005179	0.227844	32	42	Up
hsa-miR-889	0.88	6.39	8.56	0.006415	0.242032	72	96	Up
hsa-miR-539-3p	0.80	3.14	8.50	0.006581	0.243816	7	10	Up
hsa-miR-708-5p	−1.45	8.83	12.76	0.001194	0.163057	592	290	Down
hsa-miR-744-5p	−0.81	7.40	11.97	0.001618	0.172186	207	122	Down
hsa-miR-3653	−0.97	2.99	9.94	0.003613	0.206878	9	5	Down
hsa-let-7g-5p	−0.64	13.97	9.91	0.003657	0.20753	18189	13575	Down
hsa-let-7b-5p	−0.71	12.52	9.17	0.004964	0.225185	7078	4459	Down
hsa-miR-664-5p	−0.87	3.25	8.85	0.005685	0.233856	11	7	Down
hsa-miR-5701	−1.02	3.59	8.42	0.006808	0.246205	13	9	Down

Controlling Age, Gender and Stage. *p* value < 0.01 and fold change > 1.5. FDR: false discovery rate; miR: miRNA; Log FC: log fold change; Log CPM: log counts per million; LCT: large cell transformation.

## Data Availability

These datasets are deposited in the Gene Expression Omnibus (GEO) repository under the accession numbers GSE181130 and GSE113113.

## Supplementary Materials

The following are available online at https://www.mdpi.com/article/10.3390/cancers13225854/s1, Figure S1: The distribution of various immune cell sub-types in LCT-MF and non-LCT. A. Profiling tumor infiltrating immune cells with CIBRSORT. *p* values < 0.05 were considered statistically significant. B. PD1 and PD-L1 expression on immune cells were profiled using CIBRSORT in the tumor microenvironment of MF-LCT and non-LCT.
