# Supplementary material for: Identification of a Distinct miRNA Regulatory Network in the Tumor Microenvironment of Transformed Mycosis Fungoides
Source: Cancers (Basel). 2021 Nov 22;13(22):5854. doi: 10.3390/cancers13225854 (PMC8616450; doi:10.3390/cancers13225854)
Supplement: Supplementary file 1 [file cancers-13-05854-s001.zip › cancers-1453547-supplementary.pdf]

cancers

MDPI

Article

Identification of A Distinct miRNA Regulatory Network in the Tumor Microenvironment of Transformed Mycosis Fungoides

Cosimo Di Raimondo, Zhen Han, Chingyu Su, Xiwei Wu, Hanjun Qin, James F. Sanchez, Yate-Ching Yuan, Xochiquetzal Martinez, Farah Abdulla, Jasmine Zain, Chun-Wei Chen, Steven T. Rosen and Christiane Querfeld

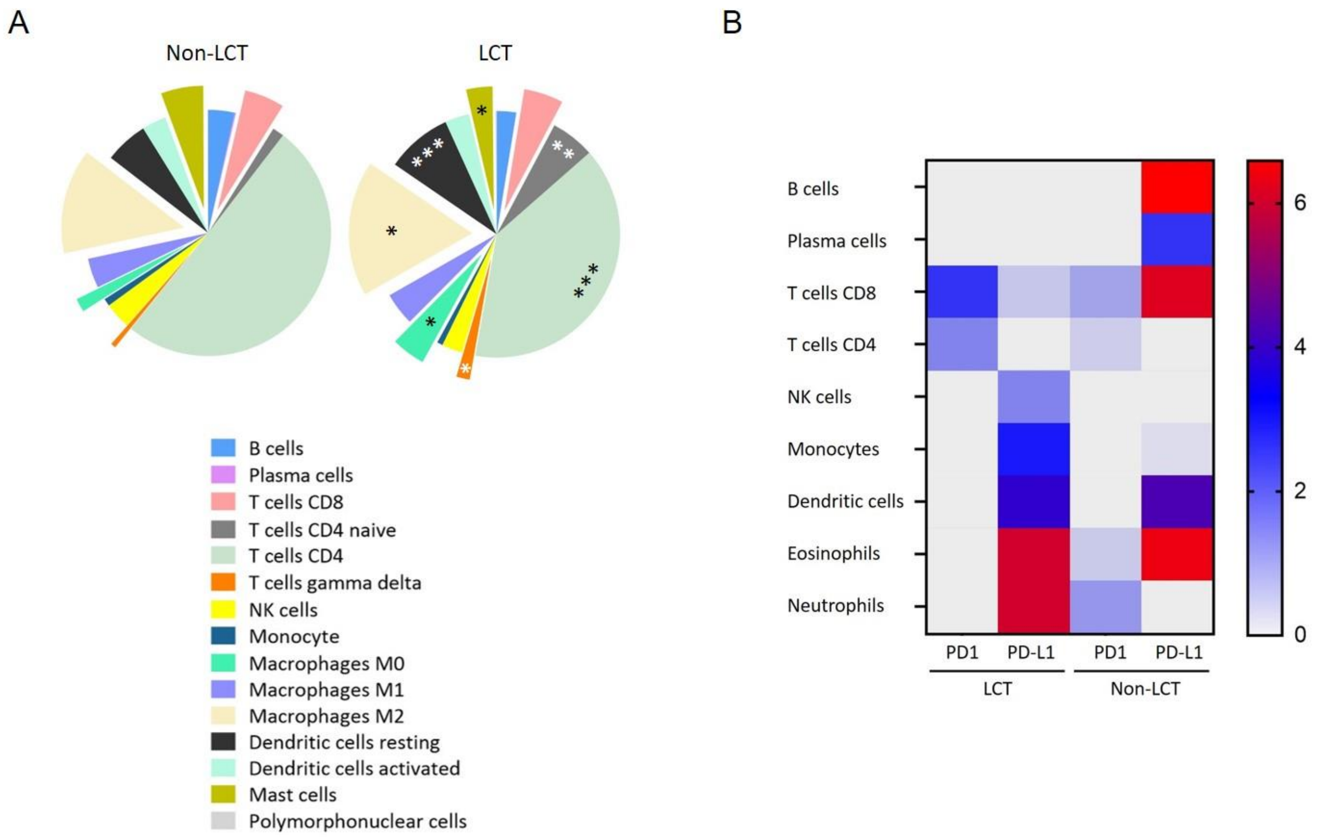

Figure S1. The distribution of various immune cell sub-types in LCT-MF and non-LCT. **A.** Profiling tumor infiltrating immune cells with CIBRSORT. *P* values < 0.05 were considered statistically significant. **B.** PD1 and PD-L1 expression on immune cells were profiled using CIBRSORT in the tumor microenvironment of MF-LCT and non-LCT.
